# Spatial inhibition of return is impaired in mild cognitive impairment and mild Alzheimer’s disease

**DOI:** 10.1371/journal.pone.0252958

**Published:** 2021-06-14

**Authors:** Xiong Jiang, James H. Howard, G. William Rebeck, Raymond Scott Turner

**Affiliations:** 1 Department of Neuroscience, Georgetown University Medical Center, Washington, D.C., United States of America; 2 Department of Psychology, The Catholic University of America, Washington, D.C., United States of America; 3 Department of Neurology, Georgetown University Medical Center, Washington, D.C., United States of America; Istituto Di Ricerche Farmacologiche Mario Negri, ITALY

## Abstract

Spatial inhibition of return (IOR) refers to the phenomenon by which individuals are slower to respond to stimuli appearing at a previously cued location compared to un-cued locations. Here with a group of older adults (n = 56, 58–80 (67.9±5.2) year old, 31 females, 18.7±3.6 years of education), we provide evidence supporting the notion that spatial IOR is mildly impaired in individuals with mild cognitive impairment (MCI) or mild Alzheimer’s disease (AD), and the impairment is detectable using a double cue paradigm. Furthermore, reduced spatial IOR in high-risk healthy older individuals is associated with reduced memory and other neurocognitive task performance, suggesting that the double cue spatial IOR paradigm may be useful in detecting MCI and early AD.

## Introduction

Spatial inhibition of return (IOR) refers to the phenomenon by which individuals are slower to respond to stimuli appearing at a previously cued location compared to un-cued locations [1]. In a classic spatial cue-target paradigm, subjects are usually faster to respond to the target appearing at the cued than un-cued location when the stimuli onset asynchrony (SOA) between the target and cue is short (~200ms or less), but are slower when the SOA is long (~300-500ms or more), with the latter generally referred as spatial IOR. First reported in 1984 [2], spatial IOR has been studied using different modalities of stimuli [3], different responses (i.e., manual vs. saccadic response) [4, 5], and in older adults [6].

In addition to the superior colliculus [7], cortical areas such as the temporoparietal junction (TPJ) and the inferior parietal cortex have been shown to play critical roles in spatial IOR [8]. Given that these two regions are involved in AD progression [9], this suggests that spatial IOR may be useful in assisting MCI and AD diagnosis. However, it remains controversial whether spatial IOR is impaired in AD. Early studies suggest that spatial IOR is relatively preserved in AD [10–12]. By contrast, more recent studies suggest that spatial IOR may be impaired in individuals with MCI or AD [13–15], and IOR deficits in individuals with MCI may be predictive of conversion to dementia [15]. Most previous studies employed the classic single cue-target paradigm [2].

Previous studies have suggested that increasing the number of cues may lead to an increase in IOR effect [16, 17]. We hypothesized that spatial IOR deficits were present in individuals with MCI or mild AD, and the deficits could be detected using a double cue spatial IOR experimental design.

## Materials and methods

### Participants

Eight individuals with MCI, seven individuals with mild AD, and 41 healthy older adults participated in the study (Table 1). All MCI and mild AD participants were recruited from the Memory Disorders Program (MDP) (under the supervision of R.S.T.) at Georgetown University Medical Center. Healthy controls were recruited through flyers at local communities, online and newspaper advertisement, and flyers at the MDP and other local memory clinics. Approximately one third of control participants had at least one first-degree relative who had a diagnosis of probable AD or was suspected of AD. Prior to enrollment, a signed informed consent form approved by the Georgetown University Medical Center’s Institutional Review Board was obtained from all participants and their legally authorized representatives (if they had a diagnosis of MCI or mild AD). Data from a few additional participants were excluded from the analysis due to exclusion criteria (S1 Table), and individual data from all participants (including participants who are excluded from main data analysis) is listed in the S1 Table for reference.

**Table 1:** The demographics and neuropsychological test scores of control, MCI, and mild AD participants.

	Controls			MCI/AD			*p* ^1^	
	Low-risk	High-risk	Combined	MCI	AD	Combined	*Four Groups*	*Combined (Controls vs MCI/AD)*
N (F) ^3^	21 (10F^2^)	20 (16F^2^)	41 (26F^2^)	8 (2F^2^)	7 (3F^2^)	15 (5F^2^)	0.028	0.040
Age	68.7±5.6	66.0±4.8	67.4±5.3	71.3±3.1	67.0±5.5	69.3±4.8	n.s.	n.s.
Education (yrs)	18.5±4.0	18.4±2.8	18.4±3.4	20.5±3.5	18.3±4.9	19.5±4.2	n.s.	n.s.
%CA ^3^	71.4%	85.0%	78.1%	87.5%	85.7%	86.7%	n.s.	n.s.
*APOE4* carriers (%)^3^^,^^4^	0%	60.0%	29.3%	62.5%	57.1%	60.0%	<0.001	0.028
AD family history (%)^3^^,^^5^	0%	75.0%	36.6%	37.5%	57.1%	46.7%	<0.001	n.s.
MMSE	29.1±1.1	29.5±1.0	29.3±1.0	28.1±1.8	27.0±2.3	27.6±2.0	<0.001	0.0001
MoCA ^6^	25.1±1.9	25.3±2.0	25.2±1.8	23.2±2.4	21.0±4.9	22.1±3.8	0.043	0.0089
LM Immediate	11.0±3.2	13.2±3.5	12.0±3.5	10.1±2.4	6.1±4.3	8.3±3.9	<0.001	0.0011
LM Delayed	8.7±3.9	10.5±4.5	9.5±4.2	7.5±4.0	3.7±4.0	5.7±4.3	0.005	0.0045
LM Retention Rate (%)^7^	76.3±26.9	76.4±17.8	76.4±22.6	68.7±30.4	38.6±39.6	54.7±37.1	0.045	0.0105
ADAS-cog	7.1±3.3	5.5±3.5	6.3±3.4	13.1±4.3	16.1±7.7	14.5±6.1	<0.001	5.0E-08
NPI	2.1±4.2	2.5±5.5	2.3±4.9	4.6±3.9 ^8^	3.9±5.4	4.2±4.5	n.s.	n.s.
LADL	76.4±2.4	76.2±3.5	76.3±3.0	71.7±8.2 ^8^	72.1±8.7	71.9±8.2	n.s.	0.0054
LVF	47.2±14.6	49.2±11.7	48.2±13.1	46.3±14.7	35.4±8.3	41.2±13.0	n.s.	n.s.

ADAS-Cog, Alzheimer’s Disease Assessment Scale-Cognitive subscale; CA, Caucasian-Americans; LADL, Lawton Instrumental Activities of Daily Living Scale; LM, Logical Memory Test; LVF, Letter Verbal Fluency; MCI, mild cognitive impairment; MMSE, mini-mental state exam; MoCA, Montreal Cognitive Assessment; NPI, Neuropsychiatric Inventory.

^1^ uncorrected p values for the difference among the Four Groups (low-risk controls, high-risk controls, MCI, and AD) with one-way ANOVA (unless otherwise specified) or between controls and MCI/AD with two-sample t-tests (unless otherwise specified), all tests were two-tailed;

^2^ female, adding sex as a covariate produced similar (nearly identical) results;

^3^ Fisher’s Exact Test;

^4^ There are four homozygous APOE4 carriers in the MCI/AD group, and one in the control group;

^5^ One control who was an APOE4 carrier and had a grandmother with dementia and one AD patient whose uncle and grandparents had dementia were coded positive for AD family history, and all others who were coded positive for AD family history had first-degree relatives with AD or probable AD;

^6^ MoCA were only administered to a subset of participants, including 17 controls and 10 MCI/AD patients (out of 41 controls and 15 MCI/AD patients included here;

^7^ Retention rate was limited to 100%, and retention rate for one AD participant was set to 0 as this participant scored 0 for both immediate recall and delayed recall;

^8^ NPI and LADL is no available in one MCI participant.

### Neuropsychological and other assessments

The following data were collected from all participants: blood pressure; biographical and health questionnaire; family history of AD; Mini-Mental State Examination (MMSE); Montreal Cognitive Assessment (MoCA); Alzheimer’s Disease Assessment Scale—Cognitive subscale (ADAS-Cog); Lawton Instrumental Activities of Daily Living Scale (LADL); Neuropsychiatric Inventory (NPI); Letter Verbal Fluency (LVF); Logical Memory subtest of the Wechsler Memory Scale (WMS)–fourth edition (WMS-IV). Family history of probable Alzheimer’s disease or dementia was collected during the study visit. Saliva samples were collected from all except one AD patient for APOE genotyping, which was carried out at G.W.R.’s lab at Georgetown University. Controls were divided into two groups based on AD family history and APOE genotypes: low-risk, controls without an AD family history and with zero copy of APOE4 allele (n = 21); high-risk, controls with an AD family history and/or at least one copy of APOE4 allele (n = 20) (Table 1).

### Spatial IOR experimental design

**Fig 1:**
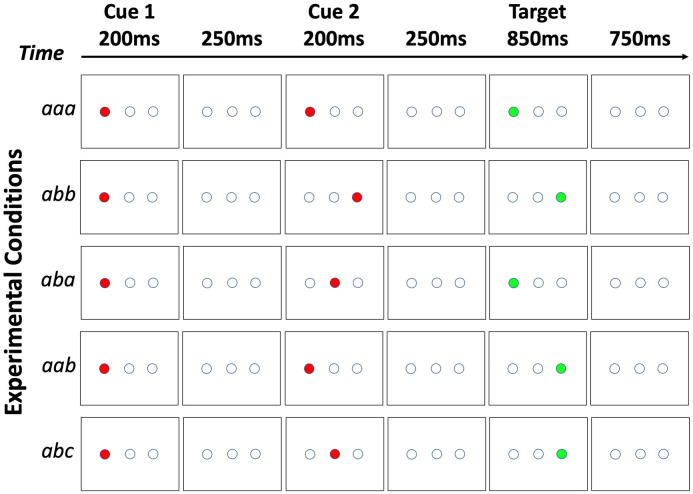
The double-cue spatial IOR experiment paradigm. Within each trial, there were three sequentially presented visual stimuli—two cues (solid red circle) and one target (solid green circle)—with a blank screen in between. The three stimuli were presented serially. The two cue stimuli could appear in any of the three locations (left, middle, right), whereas the target stimuli could only appear in one of the two locations (left or right, but not the middle). Subjects were instructed to respond to the target (solid green circle) by pressing one of two buttons in the right hand to indicate whether the target was presented at the left or right location. The two cues were presented 200msec each, with a 250msec break in between. The second cue was followed by another 250msec break before the onset of the target, which was presented for 850msec. The next trial started 750msec after the offset of the target stimulus. There were five conditions based on the relationship of the locations in which the three stimuli were presented, *aaa*, *abb*, *aba*, *aab*, and *abc* (see main text). One example of each condition is shown here (in this example the first cue always appears in the left circle, but in the actual experiment the first and the second cue could appear in any of the three locations).

The experimental paradigm was adapted from a previous learning study [18]. Within each trial, three stimuli were presented sequentially, including two cue stimuli (solid red circle) and one target stimulus (solid green circle). The subjects were instructed to observe the two red cues and respond to the green target to indicate whether the green target was presented at the left or right location by pressing one of two buttons in the right hand (with the index and the middle finger) (Fig 1). The two cue stimuli could appear for 200ms each in any of the three possible locations (left, center, right, shown as the three empty circles in Fig 1), whereas the target stimuli could only appear in two possible locations (left or right, but not center) for 850ms, then was followed by a 750ms blank screen before next trial started. Subjects had to respond within the 1.6sec time-window. Two runs of data were collected from all except 3 subjects, who only finished one run of experiment. There were 130 trials per run, and each trial lasted 2.5s. The center circle was presented at the center of the screen. The diameter of each circle was 21 pixels, and the center-to-center distance between two neighboring circles was 57 pixels. The spatial resolution of the monitor was set to 1024x768. The visual angle of each circle was approximately 0.5°. Participants were instructed to keep their fixation at the center of the screen.

There were five conditions based on the locations in which the two cues and the target were sequentially presented (see Fig 1 for an example of the five conditions).

***aaa***, in which the two cues and the target were presented at the same location;
***abb***, in which the second cue and the target were presented at the same location, and the first cue was presented at a different location;
***aba***, in which the first cue and the target were presented at the same location, and the second cue was presented at a different location;
***aab***, in which the two cues were presented at the same location, and the target was presented at a different location;
***abc***, in which the two cues and the target were presented at three different locations.

### Statistical analysis

The group (controls vs MCI/AD) difference in demographics and neuropsychological tests were investigated using two-sample t-tests or Fisher’s Exact tests. The spatial IOR effects were investigated using mixed-design ANOVAs with one within-subject factor (five experimental conditions, Fig 1), and one between-subject factor (four groups: low-risk controls, high-risk controls, MCI, and AD). The F and p values of the coefficients in the mixed model ANOVA were adjusted using the Greenhouse-Geisser correction. To account for the difference in mean RT between MCI/AD patients and controls (*p* = 0.001) so that the IOR difference can be better detected, raw RT was first normalized for each subject separately:

$$normRT=\frac{RT-meanRT}{meanRT}$$

Where RT represents the RT of each individual trial, and meanRT represents the average RT across all trials with correct response, and normRT represents normalized RT. Data analysis was then conducted on the normalized RTs (normRT). Both the raw RT and normalized RT (normRT) of each individual subject is shown in S1 Table.

### Effect size

To further assess the difference in IOR effects between controls and MCI/AD participants, we calculated and provided effect sizes for IOR effects calculated from each pair of conditions and for the four comparisons (low-risk controls versus MCI, low-risk controls versus AD, high-risk controls versus MCI, and high-risk controls versus AD). Briefly, we first computed the difference between each pair of conditions (as a measure of IOR effect), then calculated the effect sizes between controls (low- or high-risk) and patients (MCI or AD). Due to imbalanced sample size (i.e., more controls than MCI or AD patients), Hege’s *g* was used.

## Results

The demographics, *APOE4* status, AD family history, and neuropsychological tests scores of the participants are shown in Table 1.

**Fig 2:**
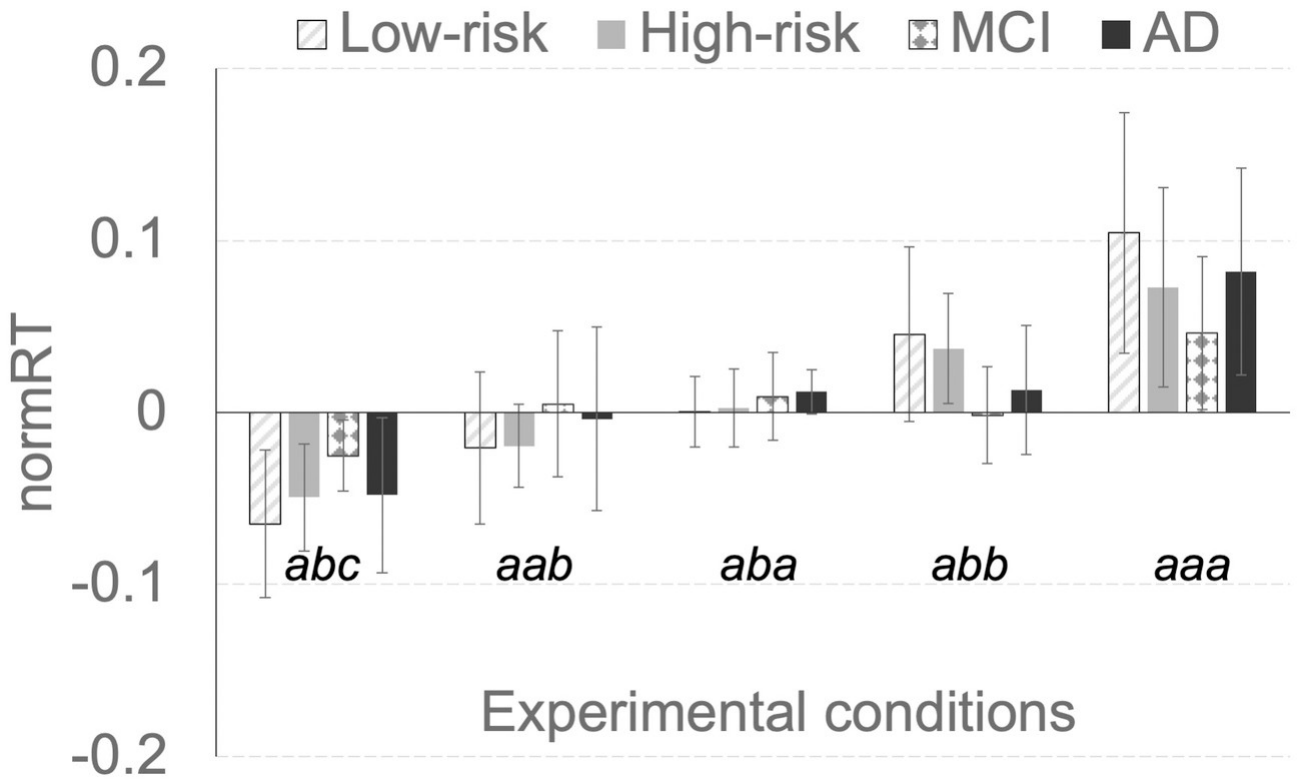
The double-cue spatial IOR experiment data. Mean normRT from 21 low risk controls, 20 high risk controls, eight MCI participants, and seven mild AD participants. Error bars represent standard deviation.

A mixed-design ANOVA with a within-subject factor (Condition: *abc*, *aab*, *aba*, *abb*, *and aaa*) and a between-subject factor (Group: low-risk controls, high-risk controls, MCI, and AD) revealed significant effects of Condition, F(4,208) = 42.666, p<0.001, and a Group x Condition interaction, F(12,208) = 2.073, p = 0.039, but not Group, F(3,52) = 1.518, p = 0.221 (Fig 2). Similar results were obtained when low- and high-risk controls were collapsed into one group (Control), and MCI and AD participants were collapsed into another group (MCI/AD): significant effects of Condition, F(4,216) = 42.017, p<0.001, and a Group (Controls vs MCI/AD) x Condition interaction, F(4,216) = 3.816, p = 0.014, but not Group, F(1,54) = 0.747, p = 0.391.

**Fig 3:**
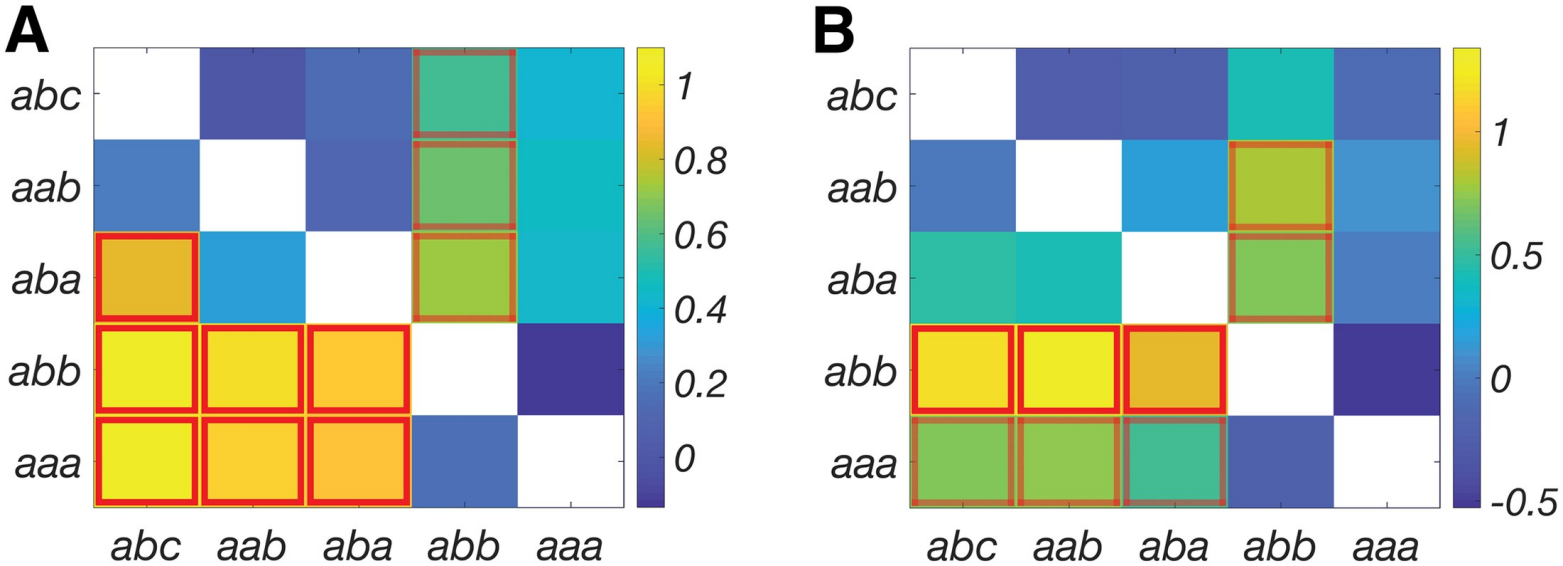
The effect sizes of difference in spatial IOR effects between low-/high-risk controls and MCI or AD participants. The IOR effects were measured as the difference between each pair of experimental conditions in Fig 1. (**A**) Low risk controls versus MCI (lower triangle) and AD (upper triangle) participants. (**B**) High risk controls versus MCI (lower triangle) and AD (upper triangle) participants. “Large” effect sizes (>0.8) are highlighted with a solid red box, and “medium” effect sizes (0.5–0.8) are highlighted with a dashed red box.

Fig 3A shows the effect sizes of differences in IOR effects (measured as the difference between each pair of conditions, i.e., normRT_*abb*_ − normRT_*aba*_) between low-risk controls and MCI participants (lower triangle), and between low-risk controls and AD participants (upper triangle). Similarly, Fig 3B shows the effect sizes of differences in IOR effects between high-risk controls and MCI participants (lower triangle) and between high-risk controls and AD participants (upper triangle). Across all four different comparisons, the effect sizes for the IOR effects measured as normRT_*abb*_ − normRT_*aba*_ and normRT_*abb*_ − normRT_*aab*_ were consistently larger than 0.5, suggesting a larger than “medium” effect to differentiate MCI or AD participants from healthy controls.

**Fig 4:**
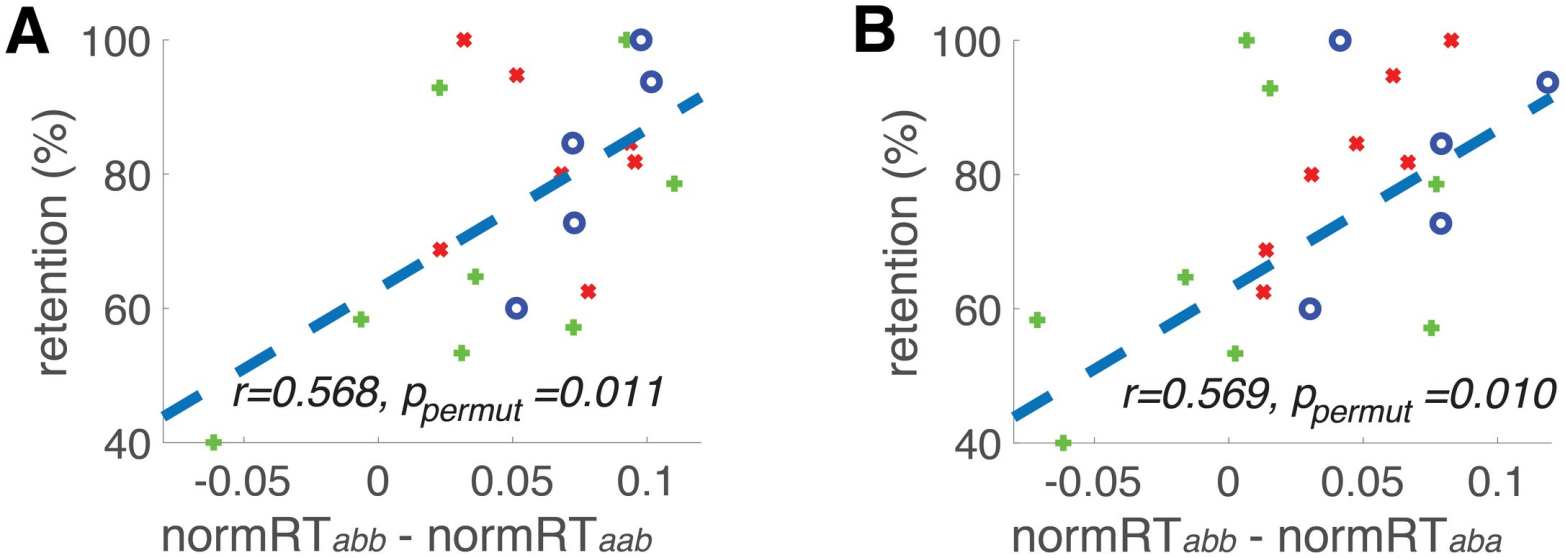
The correlation between Logical Memory (LM) subtests retention rate and two spatial IOR effects. (**A**) IOR effect was measured as the difference between condition *abb* and *aab* (*normRT*_*abb*_*—normRT*_*aab*_). (**B**) IOR effect was measured as the difference between condition *abb* and *aba* (*normRT*_*abb*_*—normRT*_*aba*_). Each marker represents one participant: o, *APOE4* non-carriers with an AD family history; +, *APOE4* carriers without an AD family history; x, *APOE4* carries with an AD family history. Error bars represent standard deviation.

Furthermore, we investigated whether the spatial IOR effects were sensitive to potential early AD pathological changes. Two IOR effects, (normRT_*abb*_ − normRT_*aba*_) and (normRT_*abb*_ − normRT_*aab*_), were used as both had larger than “medium” effect size in differentiating controls from MCI/AD patients in all four group comparisons (i.e., low- or high-risk controls versus MCI or AD, see Methods) (Fig 3). Pearson’s correlation analyses were used to examine the relationship between spatial IOR effect and four neuropsychological test scores (MMSE, LM immediate recall, LM delayed recall, and LM retention rate). Statistical significance of these correlations was determined by permutation testing with 10000 randomly shuffled samples (Table 2). Both IOR effects (normRT_*abb*_ − normRT_*aab*_ and normRT_*abb*_ − normRT_*aba*_) significantly correlated with LM retention rate (r = 0.568, *p* = 0.0090, *p*_*permutation*_ = 0.011; r = 0.569, *p* = 0.0089, *p*_*permutation*_ = 0.010, respectively) (Fig 4A and 4B). In addition, IOR effect measured via normRT_*abb*_ − normRT_*aab*_ correlated with MMSE score (*r* = 0.601, *P*_*permut*_ = 0.0284, data not shown). The correlation results should be taken with caution due to a modest sample size (n = 20) for correlation analysis.

**Table 2:** The correlations between IOR effects and MMSE, LM immediate recall, LM delayed recall, and LM retention rate scores.

	MMSE ^1^		LM ^2^ Immediate		LM Delayed		LM Retention	
IOR (normRT)	*r*	*p*_*perumt*_	*r*	*p*_*perumt*_	*r*	*p*_*perumt*_	*r*	*p*_*perumt*_
*abb-aab*	0.601	**0.028**	-0.077	0.709	0.232	0.317	0.568	**0.011**
*abb-aba*	0.475	0.057	0.200	0.386	0.426	0.053	0.569	**0.010**

Two IOR effects were investigated, one was measured as the difference between the condition *abb* and *aab* (*normRT*_*abb*_*-normRT*_*aab*_), the other as the difference between the condition *abb* and *aba* (*normRT*_*abb*_*-normRT*_*aba*_). Statistical significances were determined by permutation testing with 10000 randomly shuffled samples. MMSE, Mini-Mental State Examination; LM, the Logical Memory subtest of the Wechsler Memory Scale (WMS)–fourth edition (WMS-IV).

## Discussion

Previous studies using classic single cue-target paradigms report conflicting findings regarding spatial IOR impairment in individuals with MCI or AD. In the present study, we used double cue paradigm capable of detecting the spatial IOR effect in a “fine” resolution (see the RT profiles of five conditions in Fig 2), and observed spatial IOR impairment in individuals with MCI or mild AD. In addition, there was no difference in spatial IOR between individuals with MCI and individuals with mild AD, suggesting that spatial IOR impairment may occur at an early disease stage. This hypothesis was further supported by the data from the high-risk control subgroup, in which reduced spatial IOR effect correlated with reduced neuropsychological test scores, including delayed recall. The average age of those 20 high-risk controls was 66.0, suggesting that spatial IOR reduction could be an early sign of underlying pathogenesis before the onset of cognitive impairment.

In AD, in addition to the medial temporal lobe (MTL), injury to the temporoparietal association cortex is frequently observed at early disease stages [9, 19]. For instance, amyloid plaques typically appear in the posterior association cortices prior to the MTL, and metabolic dysfunction is most frequently reported in the temporoparietal regions, which has a high accuracy in assisting AD diagnosis [20]. Neural injury in the temporoparietal regions is also predictive of conversion from MCI to AD [21]. Therefore, behavioral tests focused on temporoparietal and inferior parietal cortices may have the potential to assist MCI and early AD diagnosis, including the double cue spatial IOR task. Indeed, preliminary results using various machine learning algorithms suggest that integrating spatial IOR behavioral data with standard neuropsychological tests improves classification accuracy [22]. Furthermore, spatial IOR is robust and resistant to practice effect [23, 24], thus making it an appealing tool in longitudinal studies or clinical trials. Inhibitory deficit has been detected in MCI/AD [10], Parkinson’s disease [25, 26], vascular disease [27, 28], as well as major depressive disorder [29]. It is possible that the neural mechanisms underlying general inhibitory deficits might contribute to spatial IOR deficits in MCI and AD patients, but the precise neural mechanisms underlying spatial IOR deficits in MCI and AD patients remain an open question.

There are some limitations of this study. In the present study, we did not find a significant difference between AD and MCI groups. This could be due to two factors: *i)* the MCI participants tended to be older than AD participants in the present study (p = 0.0846); and *ii*) the sample size was small, with only eight MCI and seven AD participants. As IOR deficits in MCI patients may be predictive of conversion to dementia [15], it would be of great interest to investigate the potential difference in the double-cue IOR deficits between MCI and AD patients in future studies with a large sample size. The relatively small sample size also limited our interpretation of the results, and large study could more thoroughly test those associations, especially the correlation between IOR effects and neurocognitive performance in “high-risk” controls. It would be interesting to investigate whether the difference between the conditions *aba* and *abb* is related to the difference in the IOR onset time [30]. The difference in RT among the five conditions in control is a novel finding (i.e., the difference between the *aab* and *abc* conditions) (Fig 2). We hypothesize the RT profile in controls may reflect a general disruption to the information processing circuitry, i.e., an “inhibitory” cueing effect. However, additional studies are need to verify and further investigate the double cue spatial IOR effect using different experimental designs (with different SOAs or stimuli), i.e., whether the double-cue spatial IOR effect is driven by alterations (induced by “inhibitory” cues) at an early or late stage of the information processing pathway, or both [31]. In addition, the neural mechanisms underlying the double cue spatial IOR impairment remain to be elucidated [8, 32], and longitudinal studies are required to examine whether the reduced spatial IOR effect in high-risk control individuals predicts progression to MCI or AD.

In conclusion, these findings support the notion that spatial IOR is impaired in individuals with MCI or mild AD, and the impairment is mild but detectable using the double cue paradigm implemented in the present study. In addition, data from MCI/AD patients and high-risk controls suggest that spatial IOR impairment may occur at an early disease stage.

## Supporting information

S1 Table Data from each individual participant. The data from each individual subject of the entire study sample (including subjects who were excluded from the data analysis). Data from 9 AD, 4 MCI, and 9 control subjects were excluded from the data analysis in the main article due to following exclusion criteria: ^1^younger than 58 (n = 7); ^2^older than 80 (n = 7); ^3^no high school diploma (n = 2); ^4^with HIV-disease (n = 1), ^5^failed to perform the spatial IOR task (with an accuracy less than 75% due to failure to respond within the time window) (n = 5). Several subjects met multiple exclusion criteria, but were only counted once here (see the table below for the complete list). AD, Alzheimer’s disease; MCI, mild cognitive impairment; ms, millisecond; NAN, not-a-number, i.e., data is not available because there were no correct trials; normRT, normalized RT, see the equation in the main article.
(PDF)
