# Supplementary material for: Spatial inhibition of return is impaired in mild cognitive impairment and mild Alzheimer’s disease
Source: PLoS One. 2021 Jun 14;16(6):e0252958. doi: 10.1371/journal.pone.0252958 (PMC8202934; doi:10.1371/journal.pone.0252958)
Supplement: S1 Table — The data from each individual subject of the entire study sample (including subjects who were excluded from the data analysis). Data from 9 AD, 4 MCI, and 9 control subjects were excluded from the data analysis in the main article due to following exclusion criteria: 1younger than 58 (n = 7); 2older than 80 (n = 7); 3no high school diploma (n = 2); 4with HIV-disease (n = 1), 5failed to perform the spatial IOR task (with an accuracy less than 75% due to failure to respond within the time window) (n = 5). Several subjects met multiple exclusion criteria, but were only counted once here (see the table below for the complete list). AD, Alzheimer’s disease; MCI, mild cognitive impairment; ms, millisecond; NAN, not-a-number, i.e., data is not available because there were no correct trials; normRT, normalized RT, see the equation in the main article. (PDF) [file pone.0252958.s001.pdf]

S1 Table. The data from each individual subject of the entire study sample (including subjects who were excluded from the data analysis). Data from 9 AD, 4 MCI, and 9 control subjects were excluded from the data analysis in the main article due to following exclusion criteria: [1]younger than 58 (n=7); [2]older than 80 (n=7); [3]no high school diploma (n=2); [4]with HIV-disease (n=1), [5]failed to perform the spatial IOR task (with an accuracy less than 75% due to failure to respond within the time window) (n=5). Several subjects met multiple exclusion criteria, but were only counted once here (see the table below for the complete list). *AD, Alzheimer's disease; LM, Logical Memory Test; LM retention, delayed / immediate, if both were zeros, then retention was set to zero; MCI, mild cognitive impairment; MMSE, mini-mental state exam; ms, millisecond; NAN, not-a-number, i.e., data is not available because there were no correct trials; normRT, normalized RT, see the equation in the main article (numbers represent normRT*10 to better fit within the table).*

		raw RT					normRT					Neuropsychological test scores			
Included Subjs	Accuracy (%)	abc	aab	aba	abb	aaa	abc	aab	aba	abb	aaa	MMSE	LM immediate	LM delayed	LM retention
Control	99.2	380	445	443	477	473	-1.32	0.16	0.1	0.89	0.8	30	11	8	0.73
Control	100	466	487	485	522	547	-0.59	-0.17	-0.19	0.56	1.06	29	7	4	0.57
Control	100	539	536	540	549	544	-0.05	-0.1	-0.02	0.13	0.04	30	14	13	0.93
Control	99.2	488	528	544	580	581	-0.95	-0.2	0.08	0.75	0.77	30	11	9	0.82
Control	98.1	498	481	509	531	544	-0.22	-0.56	0	0.41	0.67	29	14	14	1.00
Control	100	470	492	502	478	564	-0.42	0.02	0.21	-0.27	1.49	29	6	8	1.33
Control	98.5	591	640	615	619	631	-0.4	0.4	-0.01	0.06	0.25	30	14	11	0.79
Control	92.3	476	487	484	523	575	-0.48	-0.25	-0.32	0.47	1.5	30	13	11	0.85
Control	99.6	543	565	603	611	612	-0.7	-0.32	0.33	0.46	0.48	30	8	5	0.63
Control	99.6	525	503	537	500	535	0.12	-0.3	0.35	-0.36	0.31	29	12	7	0.58
Control	100	564	572	582	615	661	-0.45	-0.31	-0.14	0.42	1.2	28	15	12	0.80
Control	100	341	401	387	418	470	-1.3	0.22	-0.12	0.65	1.99	30	13	7	0.54
Control	99.6	442	473	450	488	509	-0.51	0.16	-0.35	0.48	0.92	30	21	21	1.00
Control	97.7	397	413	423	462	454	-0.69	-0.33	-0.08	0.82	0.64	27	12	8	0.67
Control	97.7	582	636	636	598	614	-0.49	0.39	0.39	-0.22	0.04	26	10	4	0.40
Control	88.1	376	463	435	503	479	-1.54	0.4	-0.22	1.32	0.76	30	10	11	1.10
Control	99.2	473	503	498	529	600	-0.7	-0.11	-0.2	0.41	1.81	29	19	18	0.95
Control	100	537	544	565	582	657	-0.51	-0.39	-0.02	0.29	1.61	30	10	8	0.80
Control	99.6	478	474	508	525	534	-0.46	-0.54	0.14	0.49	0.66	30	7	3	0.43

Control	98.8	511	475	503	485	561	0.21	-0.52	0.04	-0.3	1.2	29	14	13	0.93
Control	99.2	389	398	417	437	458	-0.61	-0.41	0.05	0.53	1.05	30	13	11	0.85
Control	95	284	286	301	327	355	-0.69	-0.63	-0.16	0.71	1.64	30	10	2	0.20
Control	98.1	466	476	503	529	544	-0.65	-0.45	0.08	0.6	0.9	30	12	9	0.75
Control	98.8	348	371	373	378	400	-0.6	0.02	0.1	0.23	0.81	29	12	12	1.00
Control	97.7	368	386	420	444	511	-1.12	-0.69	0.13	0.71	2.32	30	14	13	0.93
Control	100	422	436	429	481	441	-0.46	-0.14	-0.31	0.88	-0.04	30	16	15	0.94
Control	96.9	493	469	529	609	641	-0.83	-1.27	-0.17	1.33	1.91	28	13	7	0.54
Control	99.2	493	494	508	510	492	-0.17	-0.14	0.15	0.17	-0.17	28	15	8	0.53
Control	99.2	428	424	439	473	479	-0.39	-0.48	-0.15	0.62	0.77	29	14	11	0.79
Control	97.7	440	459	472	471	534	-0.57	-0.17	0.12	0.09	1.43	28	8	6	0.75
Control	99.6	445	443	483	486	497	-0.5	-0.55	0.31	0.38	0.6	30	12	14	1.17
Control	100	426	428	425	403	445	0.1	0.15	0.06	-0.44	0.55	30	12	15	1.25
Control	99.6	565	578	583	591	579	-0.24	-0.03	0.07	0.2	-0.01	30	16	11	0.69
Control	98.8	575	605	595	634	614	-0.46	0.03	-0.13	0.51	0.19	30	11	1	0.09
Control	98.5	387	389	416	408	420	-0.39	-0.33	0.34	0.13	0.42	30	8	8	1.00
Control	99.6	556	564	595	585	645	-0.45	-0.29	0.23	0.07	1.09	30	17	11	0.65
Control	98.5	484	510	521	537	585	-0.67	-0.18	0.04	0.34	1.27	30	10	6	0.60
Control	97.3	444	466	479	510	494	-0.7	-0.23	0.05	0.7	0.35	27	17	14	0.82
Control	100	369	388	416	469	473	-1.15	-0.7	-0.02	1.24	1.34	29	5	6	1.20
Control	100	483	504	521	499	489	-0.35	0.08	0.42	-0.04	-0.23	28	7	8	1.14
Control	98.8	398	473	427	477	545	-1.15	0.52	-0.51	0.61	2.12	30	10	8	0.80
MCI	100	527	546	581	546	634	-0.53	-0.2	0.43	-0.19	1.39	26	6	8	1.33
MCI	94.6	563	627	577	595	621	-0.46	0.62	-0.22	0.09	0.52	26	8	3	0.38
MCI	95.8	524	522	544	500	544	0	-0.05	0.38	-0.46	0.38	29	10	8	0.80
MCI	99.6	468	508	492	470	514	-0.36	0.48	0.15	-0.3	0.59	27	11	7	0.64
MCI	88.5	567	601	585	589	569	-0.27	0.32	0.04	0.11	-0.22	30	10	12	1.20
MCI	97.7	634	604	667	679	676	-0.27	-0.72	0.23	0.41	0.37	30	13	5	0.38
MCI	100	555	563	565	573	584	-0.19	-0.05	-0.01	0.13	0.32	27	10	3	0.30
MCI	97.7	581	578	563	582	598	0.06	-0.01	-0.26	0.08	0.35	30	13	14	1.08
AD	97.7	462	552	523	535	617	-1.16	0.55	0.01	0.24	1.79	27	6	3	0.50
AD	93.5	494	515	534	581	554	-0.74	-0.35	0.01	0.89	0.37	29	8	10	1.25
AD	96.9	442	494	476	459	472	-0.52	0.6	0.21	-0.14	0.13	29	13	7	0.54
AD	99.2	643	614	675	663	668	-0.15	-0.6	0.34	0.15	0.23	29	9	6	0.67
AD	94.6	519	589	571	572	626	-0.82	0.42	0.1	0.11	1.07	24	0	0	0.00
AD	88.5	477	453	476	472	526	0.02	-0.48	0	-0.1	1.04	27	4	0	0.00
AD	87.7	556	534	567	544	618	-0.01	-0.41	0.18	-0.23	1.1	24	3	0	0.00

		raw RT					normRT					Neuropsychological test scores			
Excluded Subjs	Accuracy (%)	abc	aab	aba	abb	aaa	abc	aab	aba	abb	aaa	MMSE	LM immediate	LM delayed	LM retention
[1]Control	99.2	380	445	443	477	473	-1.32	0.16	0.1	0.89	0.8	30	11	8	0.73
[1]Control	100	466	487	485	522	547	-0.59	-0.17	-0.19	0.56	1.06	29	7	4	0.57
[1]Control	100	539	536	540	549	544	-0.05	-0.1	-0.02	0.13	0.04	30	14	13	0.93
[1]Control	99.2	488	528	544	580	581	-0.95	-0.2	0.08	0.75	0.77	30	11	9	0.82
[1]Control	98.1	498	481	509	531	544	-0.22	-0.56	0	0.41	0.67	29	14	14	1.00
[3]Control	100	470	492	502	478	564	-0.42	0.02	0.21	-0.27	1.49	29	6	8	1.33
[1]Control	98.5	591	640	615	619	631	-0.4	0.4	-0.01	0.06	0.25	30	14	11	0.79
[1]Control	92.3	476	487	484	523	575	-0.48	-0.25	-0.32	0.47	1.5	30	13	11	0.85
[3]Control	99.6	543	565	603	611	612	-0.7	-0.32	0.33	0.46	0.48	30	8	5	0.63
[2,5]MCI	99.6	525	503	537	500	535	0.12	-0.3	0.35	-0.36	0.31	29	12	7	0.58
[2,5]MCI	100	564	572	582	615	661	-0.45	-0.31	-0.14	0.42	1.2	28	15	12	0.80
[2,5]MCI	100	341	401	387	418	470	-1.3	0.22	-0.12	0.65	1.99	30	13	7	0.54

[2,5]MCI	99.6	442	473	450	488	509	-0.51	0.16	-0.35	0.48	0.92	30	21	21	1.00
[5]AD	97.7	397	413	423	462	454	-0.69	-0.33	-0.08	0.82	0.64	27	12	8	0.67
[5]AD	97.7	582	636	636	598	614	-0.49	0.39	0.39	-0.22	0.04	26	10	4	0.40
[5]AD	88.1	376	463	435	503	479	-1.54	0.4	-0.22	1.32	0.76	30	10	11	1.10
[2]AD	99.2	473	503	498	529	600	-0.7	-0.11	-0.2	0.41	1.81	29	19	18	0.95
[2,5]AD	100	537	544	565	582	657	-0.51	-0.39	-0.02	0.29	1.61	30	10	8	0.80
[5]AD	99.6	478	474	508	525	534	-0.46	-0.54	0.14	0.49	0.66	30	7	3	0.43
[2]AD	98.8	511	475	503	485	561	0.21	-0.52	0.04	-0.3	1.2	29	14	13	0.93
[5]AD	99.2	389	398	417	437	458	-0.61	-0.41	0.05	0.53	1.05	30	13	11	0.85
[4,5]AD	95	284	286	301	327	355	-0.69	-0.63	-0.16	0.71	1.64	30	10	2	0.20
